# Efficiency Analysis of Competing Tests for Finding Differentially Expressed Genes in Lung Adenocarcinoma

**DOI:** 10.4137/cin.s791

**Published:** 2008-07-14

**Authors:** Rick Jordan, Satish Patel, Hai Hu, James Lyons-Weiler

**Affiliations:** 1 Department of Biomedical Informatics, University of Pittsburgh, Pittsburgh, PA 15260; 2 Bioinformatics Analysis Core, University of Pittsburgh, Pittsburgh, PA 15260; 3 Center for Bioinformatics, National Cancer Institute, Rockville, MD 20852; 4 Windber Research Institute, Windber, PA 15963; 5 Department of Pathology and University of Pittsburgh Cancer Institute, University of Pittsburgh, Pittsburgh, PA 15260

## Abstract

In this study, we introduce and use Efficiency Analysis to compare differences in the apparent internal and external consistency of competing normalization methods and tests for identifying differentially expressed genes. Using publicly available data, two lung adenocarcinoma datasets were analyzed using caGEDA (http://bioinformatics2.pitt.edu/GE2/GEDA.html) to measure the degree of differential expression of genes existing between two populations. The datasets were randomly split into at least two subsets, each analyzed for differentially expressed genes between the two sample groups, and the gene lists compared for overlapping genes. Efficiency Analysis is an intuitive method that compares the differences in the percentage of overlap of genes from two or more data subsets, found by the same test over a range of testing methods. Tests that yield consistent gene lists across independently analyzed splits are preferred to those that yield less consistent inferences. For example, a method that exhibits 50% overlap in the 100 top genes from two studies should be preferred to a method that exhibits 5% overlap in the top 100 genes. The same procedure was performed using all available normalization and transformation methods that are available through caGEDA. The ‘best’ test was then further evaluated using internal cross-validation to estimate generalizable sample classification errors using a Naïve Bayes classification algorithm. A novel test, termed D1 (a derivative of the J5 test) was found to be the most consistent, and to exhibit the lowest overall classification error, and highest sensitivity and specificity. The D1 test relaxes the assumption that few genes are differentially expressed. Efficiency Analysis can be misleading if the tests exhibit a bias in any particular dimension (e.g. expression intensity); we therefore explored intensity-scaled and segmented J5 tests using data in which all genes are scaled to share the same intensity distribution range. Efficiency Analysis correctly predicted the ‘best’ test and normalization method using the Beer dataset and also performed well with the Bhattacharjee dataset based on both efficiency and classification accuracy criteria.

## Introduction

Cancer research has generated a rich and complex body of knowledge, revealing cancer to be a disease involving dynamic changes in the genome.^1^ Research over the past decades has revealed a number of molecular, biochemical, and cellular traits shared by most and perhaps all types of human cancer.^2^ However, today there is a need to look at all cancers from different perspectives, using genomic^1^,^3^ and proteomic^4^,^5^ molecular techniques and try to achieve results in ways that never seemed possible.^6^

Lung cancer remains the leading cause of cancer death in industrialized countries, claiming more than 150,000 lives annually in the US.^7^ The overall 10-year survival rate is a staggering 8%–10%,^8^ and it is currently impossible to identify the high-risk patients.^9^ Today, lung carcinoma classification is based on clinicopathology. The subclassification of lung cancers is very challenging, and studies have shown that different pathologists have differed on subclassification of the same carcinomas more than half of the time.^10^ Determining metastasis of non-lung origin from lung adenocarcinomas is also difficult.^11^,^12^ A broader basis of the molecular biology of lung carcinomas could help aid in prediction of outcome, choice of therapies, and identification of new biomarkers.^13^

Microarray technology has made it possible for researchers to search for molecular markers of most cancers.^14^–^20^ A number of lung cancer studies have settled upon variable numbers in different sets of genes for successful prognostic classifiers, including Xi et al.^21^ (318 genes), Lu et al.^22^ (64 genes), Sun et al.^23^ (50 genes), Jiang et al.^24^ (36 genes), Bianchi et al.^25^ (10 genes), Chen et al.^26^ (5 genes), and Lau et al.^27^ (3 genes). The resulting classifiers may be used in all areas of prediction such as detection, choice of therapy, metastasis, and survival outcomes. While many of these models may exhibit very good accuracy, none have been clinically implemented.

By making their datasets publicly available, researchers provide opportunities for re-analysis of their data by others, including the opportunity for the comparison of a variety of distinct approaches to analysis. New methods of analysis can be developed and evaluated, hopefully leading to improvements in our understanding of the differences in the performance of distinct methods. In this study, we have re-analyzed the publicly available lung adenocarcinoma datasets of Beer et al.^9^ and Bhattacharjee et al.^13^ (both extensively studied at CAMDA 2003 conference; http://www.camda.duke.edu/camda03.html). Additionally, Efficiency Analysis was also performed using data from the Guo et al.^28^ rat toxicogenomic study data (MAQC Project). We will not try to duplicate or scrutinize their results, but rather use their data to assess a variety of analysis methods.

It is important to mention that the word ‘efficiency’ is used in statistics for describing the relative size of the variance of an estimator to a fully efficient estimator like the maximum likelihood. We introduce the concept of an empirical method to study efficiency through the consistency of statistical inferences made with any estimator. The term here is used in a slightly more generic manner as the relative internal consistency at a particular sample size. Methods that show higher internal consistency at a small *N* have more apparent power, and are therefore more efficient because they require lower *N* to achieve the same or higher observed degree of internal consistency compared to methods that require larger *N*.

## Materials and Methods

### Data retrieval

Portions of both datasets, Beer, et al. and Bhattacharjee, et al. are already available on the caGEDA website (http://bioinformatics2.pitt.edu/GE2/GEDA.html),^29^ and the data can also be obtained by links from the subsequent journal articles.^9^,^13^ Each dataset contained 5377 genes. The Beer dataset used contains data from 69 neoplastic lung adenocarcinoma samples and 17 non-neoplastic samples. The Bhattacharjee dataset used contained 52 neoplastic and 17 non-neoplastic samples. The datasets used either the Affymetrix HUGeneFL or the HG_U95Av2 microarray chip platforms, respectively. Probe sets were merged by joining Unigene cluster ID’s.

### Data and statistical analysis

All test, normalization, and transformation analyses were performed using caGEDA, a freely available informatics tool. The datasets were analyzed for differentially expressed genes (DE genes). Efficiency Analysis was performed followed by Random Resampling Validation (RRV) using a Naïve Bayes Classifier, and PACE Analysis (all described below).

### Efficiency analysis

**Figure 1:**
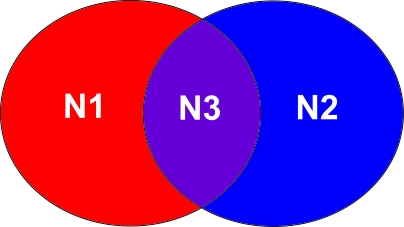
Venn diagram showing populations **N1** and **N2**, and the overlapping genes, **N3**.

**Figure 2:**
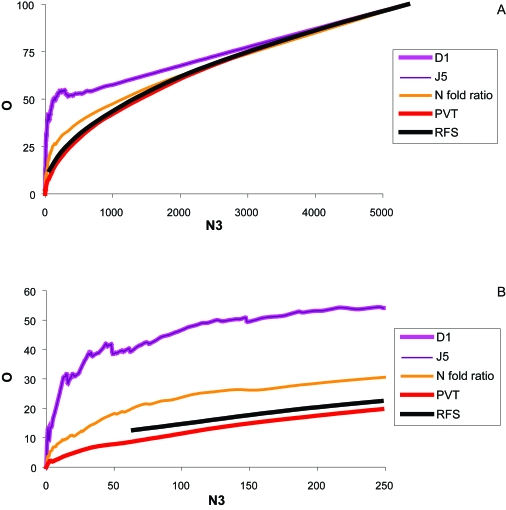
Efficiency plot of statistical tests performed without normalization comparing the Beer and Bhattacharjee datasets **(A)** Over the entire gene range, and **B**) a gene range of 0–250. The J5 and D1 far outperform others based on amount of overlap. The above is a comparison of O (% overlap) vs. N3 (number of genes). The results are from overlap of the Beer and Bhattacharjee significant gene lists. **Abbreviations:** N fold Ratio: Ratio of Mean; PVT: Pooled Variance t Test; RFS: Random Feature Selection.

Efficiency Analysis is a new method for comparing the apparent internal, or external, consistency in the list of genes found by competing methods for feature selection. In its current application, Efficiency Analysis is implemented as a method that compares the differences in the percentage of overlap of two or more methods found at the same numerical index (number) of overlapping genes, in randomly split datasets. For example, a method that exhibits 50% overlap in the 100 top genes from two studies should be preferred to a method that exhibits 5% overlap in the top 100 genes. For any test (using the same starting list), the lists overlap is 100% when all genes are included (no feature selection), assuming the same chip content. As one applies increasing stringency to the criterion for feature selection to two datasets, the number of genes that are retained decreases, and therefore the number that overlap between the two lists decreases. For any method that is less than perfectly consistent, the proportion of overlap decays as well (as the number of genes approaches zero). Methods exhibiting the highest percent overlap, at a given threshold of a filter or test, (which defines that number of genes in the observer overlap), are considered the most efficient. For internal Efficiency Analysis, a dataset of *N* samples is split into *n* non-overlapping sets, each with *N*/*n* samples. For external Efficiency Analysis, two (or more) independent datasets are generated that address the same biological or clinical question. In either case (internal or external), a given test T (t-, F-, fold-change, etc) is applied to all genes in each dataset separately. The threshold (cut-point) associated with significance level is increased in a stepwise manner over the range of significance levels (thresholds) for the test in *n* datasets. The percent overlap (O) in the independently determined ranked gene lists is determined at each threshold value (O is the relative size of the area of the overlap to the non-overlap in a Venn diagram (Fig. 1). N1 and N2 are the number of genes found to be significant at a given threshold value in dataset 1 and 2, respectively, and N3 is the number of genes in the intersection of the datasets (size of overlap, then O = N3/(N1 + N2 − N3). Plotting O VS. N3 creates an ‘efficiency curve’ for a given test (Fig. 2). For all values of N3≪M (the total number of genes), the test (t-, F-, fold-change, etc.) with the highest O at a given N3 is said to be the most efficient. For any given test, the threshold associated with N3≪M associated with the highest O is speculated to be optimal for that test. This approach does not attempt to estimate the false discovery rate (FDR; expected ratio of false positives among the significant results) but instead uses empirical consistency to guide researchers in the selection of competing diverse methods. The curve also may point to a test-threshold that exhibits increased local efficiency.

Initially, each dataset was split randomly into four representative groups; with each containing at least four normal samples and with one of the four groups containing five normals. Internal Efficiency Analysis was performed within each dataset using the Efficiency Analysis option in caGEDA (‘internal Efficiency Analysis’). For all of the tests described in this paper, external Efficiency Analysis was also performed using completely independent datasets (‘external Efficiency Analysis’). External efficiency curves were generated by pasting the ranked gene lists, with their associated test scores, into the Overlap4 tool http://bioinformatics.pitt.edu/GE2/Overlap4.html with a step of 0.1. The final gene list contains: 1) percentage of overlap (O) at a given threshold, for a specified number of genes (N3), and 2) for each selected gene, the percentage for degree of differential expression. As an independent check on the potential utility of the various gene lists, all tests were assessed for classification error using internal cross-validation (RRV with a 70%–30% split, performed at 100 iterations) with a Naïve Bayes classifier over the range of significance level for each test.

### Tests for differential expression

#### Lung cancer data

Detailed descriptions about the tests examined can be found on the caGEDA website (http://bioinformatics2.pitt.edu/GE2/GEDA.html)^29^ and some brief descriptions of the threshold-based tests and normalization methods are described in Appendix A. For most tests, a threshold of 0 was used for all tests so that all genes were returned with a score. Random Feature Selection (RFS) used a threshold range that spanned from 0 to the total number of genes (5377 distinct genes) to produce an appropriate and comparable result. PPST^30^ and SAM^31^ are permutation tests and were performed as such using the following settings: PPST using 100 permutations, threshold 1, and 1–99 quantiles; SAM using 100 permutations, δ of −1, and a δ′ of 0. The Segmented J5, described here for the first time, used 100 percentiles and a threshold of 0 for analysis. Any missing values were estimated using the K-Nearest Neighbor method with 3 neighbors.^32^ Non-distinct gene values were averaged. For initial assessment of the tests, no normalization/transformation method was used. For the normalizations/transformations, the best performing test within the acceptable gene number range, D1, was used to find the best accompanying normalization/transformation method.

#### Rat toxicogenomic data

Data were obtained from the MAQC Project website (http://www.fda.gov/nctr/science/centers/toxicoinformatics/maqc/). Efficiency Analysis was performed for the desired tests using caGEDA and parameters described above for internal Efficiency Analysis and the individual tests. Because the distributed data were previously normalized and filtered by the authors,^28^ additional normalizations were not performed in assessing the tests. Results from the J5 family, fold-change family, t-test, and random feature selection were compared.

### Naïve Bayes classification error estimation

Random Resampling Validation (RRV) was performed on the Beer et al. dataset with the Naïve Bayes classifier to produce internal cross-validation estimates of classifier performance characteristics. As a preliminary assessment of the variation in performance associated with different normalization methods, the D1 test was used in combination with transformation/normalization methods. The following parameters were used: Mean as a measure of central tendency, Naïve Bayes Classifier with the proportion of samples as the prior, 100 iterations, a 70%–30% training/test split, and a threshold range of 0 to 50 with a 1.0 step. The Bhattacharjee dataset threshold ranged from 0 to 1 with a step of 0.01. Unfortunately, tests that implement permutations (SAM and PPST) were not performed using RRV because caGEDA can not perform both resampling and randomization in stages simultaneously. Similar ranges and steps were used for the normalization methods, and a similar number of iterations were used in choosing the steps for the exceptions. The optimal threshold value was used for the classification of the initial training/test set. The selected gene list, under the most efficient normalization and test combination, was used to predict sample class labels using the Naïve Bayes Classifier. Importantly, reciprocal external cross-validation was performed for each data set i.e. one dataset was used for training, and then tested on the other, and vice-versa.

### Pace analysis

Additional computational validation was performed using PACE Analysis.^33^ Permutation Achieved Classification Error (PACE) uses permutations of the given data set to determine if the achieved classification errors are significant at the 95% and 99% levels.

### Intensity-related bias

As our study proceeded, we observed an apparent signal intensity-related bias of the J5 and derived tests toward the high end of the intensity range. Other tests appear to favor genes either at the low end, or in the mid-intensity range. To evaluate the impact of this problem, and the effects of other potential intensity-related biases, we rescaled each gene by the formula:

new_value = (old_value−min_value)/(max_value−min_value) across all of the chips (within genes) and repeated Efficiency Analysis on all methods.

### J5 Test

The J5 test is a gene-specific ratio between the mean difference in expression intensity between two groups, A and B, to the average mean group difference of all M genes. The J5 is intended for use when t-tests are likely to exhibit unacceptably low specificity (high false discovery rates) due to unstable estimates of the variance.^29^ For a two-group (A v B) comparison, the J5 test is described for the *i*^th^ of m genes as:

(1) $$J5_{i}=\frac{{\bar{\text{A}}}_{i}-{\bar{\text{B}}}_{i}}{\frac{1}{m}\sum_{j-1}^{m}|{\bar{\text{A}}}_{j}-{\bar{\text{B}}}_{j}|}$$

### Segmented J5

The Segmented J5 genes are ranked by median intensity and the entire distribution is divided into quantiles (default 100). We then used the mean parameter estimates from within each quantile separately, therefore eliminating the signal intensity bias. The mean of the absolute value of the difference of means within a quantile is used for each gene as the J5 denominator for all genes in that quantile.^34^

### Intensity scaled J5

The Intensity Scaled J5 is similar to the segmented J5 but scaled using one of the mean values (in case vs. control comparisons, the control comparison).^34^

### D1 Test

The D1 test is a twice-iterated J5 test where the remaining genes are tested after removing the initially discovered significant genes during the first iteration.^29^

## Results

Because we were most interested in relative efficiency where N3≪M, we compared methods over a range N3 = 0–250 for all tests. Three separate Efficiency Analyses were performed for the lung adenocarcinoma data: 1) ‘BETWEEN’ datasets, 2) ‘BEER only’, and 3) ‘BHATTACHARJEE only’. Two classifications were performed: 1) Beer—training/test with Bhattacharjee as a validation set, and 2) Bhattacharjee—training/test with Beer as the validation set.

### Efficiency analysis

#### Best test ‘BETWEEN’ the datasets

In this exercise we compared the genelists from Beer et al. and Bhattacharjee et al. Efficiency Analysis was performed to determine the most consistent test. Under our criterion of efficiency, the D1 and J5, appeared to produce similar results over a range of <1000 genes. While apparently far superior to the other tests as well as random feature selection (Fig. 2A, Appendix B1) within the <2500 gene range, the D1 and J5 were also superior in our range of interest (0–250) (Fig. 2B, Appendix B2). Further exploration of normalization was performed using the D1 test only.

Corrections for the intensity-related bias we observed did not influence the results greatly (for example, compare J5 to Segmented (Seg J5; Appendix B3). The results showed that the Intensity Scaled J5 seemed to be most consistent over the lower range of genes, 0–2000 genes. Other tests outperformed at higher values of N3, but not until several thousands of genes were included (Appendix B1 and B3), however, the D1, J5, and Segmented J5 outperformed Random Feature Selection greatly, and appeared to be the most consistent test at a lower number of desired genes.

### Most efficient transformation/normalization methods, both datasets

**Figure 3:**
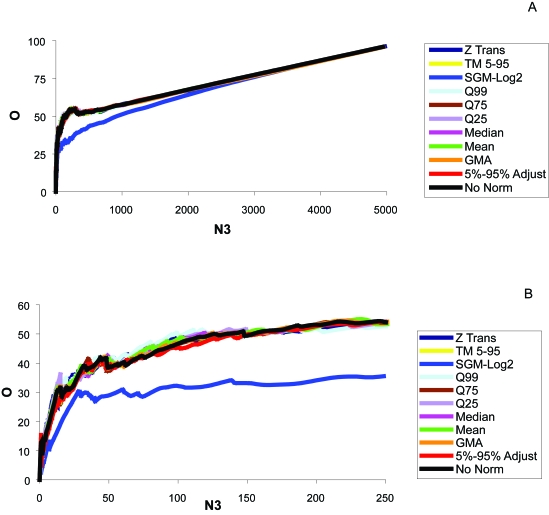
Efficiency plot of normalization and transformation methods of the Beer and Bhattacharjee datasets **A)** Over the entire gene range, and **B**) a gene range of 0–250. The D1 test was used with all methods. Again, the above results are from a comparison of O (% overlap) vs. N3 (number of genes). The results are from overlap of the Beer and Bhattacharjee significant gene lists. A close look where N3≪m (Figure 2B) reveals that a few methods actually do minimally improve the efficiency. Q: Quantile Normalization; TM: Trimmed Mean; GMA: Global Mean Adjustment; 5%–95%: Global Quantile Normalization utilizing the 5th–95th quantiles; SGM-Log2: The Subtract Global Minimum procedure was performed to eliminate negative values and allow for log transformation.

To study the effects of competing normalization effects on the efficiency of the D1 test, we generated efficiency curves for the D1 test under a variety of normalization methods. The locally optimized overlap at low N3 for the D1 test alone was 41.4% and occurred at 47 genes. Figure 3 shows the results from the normalization comparisons. Although none of the normalization methods led to marked improvement, (Appendix B4 and B5), the quantile normalizations appeared to improve overlap results slightly over our gene range (0–100). Quantiles 25, 75, and 99 all performed best at some point over the gene range. Therefore the “best” method of normalization depends on the number of genes in question. The Efficiency Analysis results from D1 alone improved minimally using these normalizations (Fig. 3).

### Most internally efficient methods, BEER dataset

**Figure 4:**
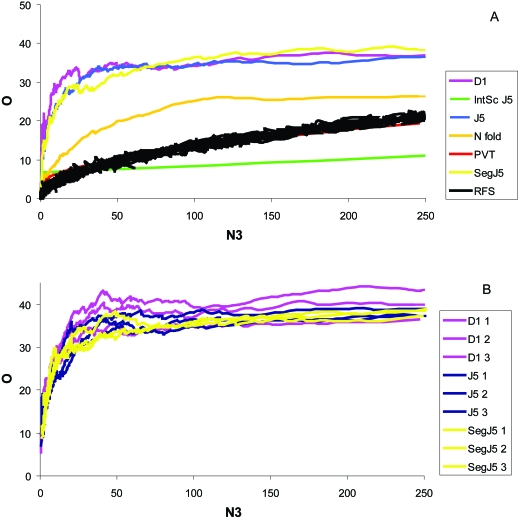
Efficiency plot of statistical tests and the three most efficient tests performed without normalization from the Beer dataset **A)** A plot of several tests over the 0–250 gene range, and **B**) a plot of the three best performing tests over the same range. Certain tests appear to be more efficient depending on the number of genes desired, but a few outperform all others over the given range. The above results are from a comparison of O (% overlap) vs. N3 (number of genes). The results are from overlap of the Beer significant gene lists. It is apparent that the most efficient test is the D1. D1 1, 2, and 3 are three iterations of the D1 test.

We split the Beer dataset randomly three times producing four independent, representative sample groups. Each group produced a gene list from the two populations (normal-cancer). As before, Efficiency Analysis was performed to determine the most efficient test. The D1, J5, and Segmented J5 all appeared to give similar results over the specified range of genes (0–250), while being far superior to the other tests as well as random feature selection (Fig. 4A, Appendix B6). To reduce the slight variation in performance associated with any arbitrary split, each of the most efficient tests were re-analyzed 3-fold (Fig. 4B). Because the D1 test was evidently the most efficient test for this dataset (independent of intensity-scaling), further exploration of normalization was performed using the D1 test only.

**Figure 5:**
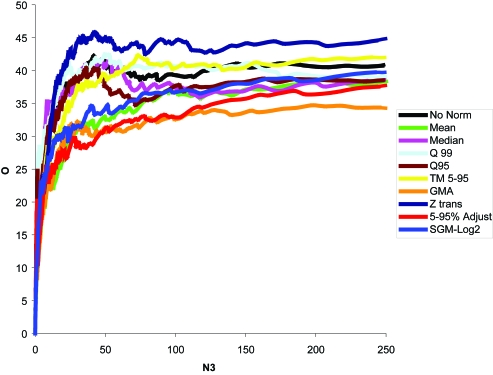
Efficiency plot of normalization and transformation methods from the Beer dataset The above results are from a comparison of O (% overlap) vs. N3 (number of genes). The results are from overlap of the Beer significant gene lists. The D1 test was used with all available methods. It is apparent that the Z transformation appeared to produce the greatest overlap over most of the given range (0–250 genes), but others appeared optimal at the extreme low end.

As before, we wanted to assess the transformation/normalization methods to see if, when used in combination with the D1 test, the percent overlap results would improve. The maximum overlap for the D1 test alone was 35% and occurred around 46 genes. The Z transform normalization method appeared to be most consistent over the entire range of genes (0–250). With Z transformation, the highest percent overlap occurred at 42 genes (Fig. 5, Appendix B7), with 21 of the genes being present on all lists 100% of the time (Appendix C, Table A). The normalization method increased O to almost 46% (Fig. 5, Appendix B7).

### Most internally efficient methods, BHATTACHARJEE dataset

**Figure 6:**
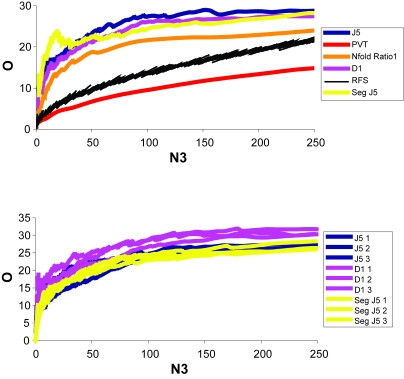
Efficiency plot of statistical tests and the three most efficient tests performed without normalization from the Bhattacharjee dataset **A)** A plot of several tests over the 0–250 gene range, and **B**) a plot of the three best performing tests over the same range. Certain tests appear to be more efficient depending on the number of genes desired, but a few outperform all others over the given range. The above results are from a comparison of O (% overlap) vs. N3 (number of genes). The results are from overlap of the Bhattacharjee significant gene lists. It is apparent that the most efficient test is the D1. D1 1, 2, and 3 are three iterations of the D1 test.

Again, Efficiency Analysis was performed to determine the most consistent test. As before, the D1, J5, and Segmented J5 all appeared to give similar results over the specified range of genes (0–125), while being superior to the other tests as well as random feature selection (Fig. 6, Appendix B8). Again, these three tests were re-analyzed at 3-fold (Fig. 6), with all three D1 tests most efficient. Next, the transformation/normalization methods were tested. The maximum overlap for the D1 test alone was 28.7% and occurred around 101 genes (~100 genes desired).

**Figure 7:**
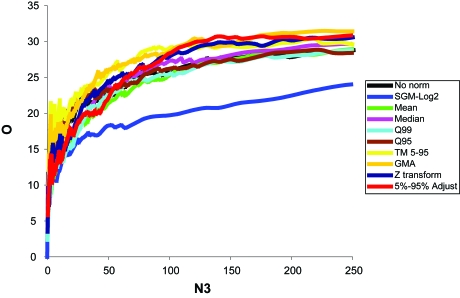
Efficiency plot of normalization and transformation methods from the Bhattacharjee dataset The above results are from a comparison of O (% overlap) vs. N3 (number of genes). The results are from overlap of the Bhattacharjee significant gene lists. The D1 test was used with all normalization methods. The Subtract Global Minimum procedure was performed to eliminate negative values and allow for log transformation. At 100 genes, TM 5%–95% appeared to be the best normalization method.

The results from the normalization comparisons are summarized as Figure 7, Appendix B9. The Trimmed mean 5%–95% normalization method outperformed all others over almost the entire range of genes (0–250). With TM 5%–95%, the highest percent overlap occurred at 102 genes, with 32 of the genes being present on all lists 100% of the time (Appendix C, Table C). The normalization method increased the O to 29.2%.

### Classification

#### Classification of the Beer dataset

##### Initial training using Beer dataset

**Figure 8:**
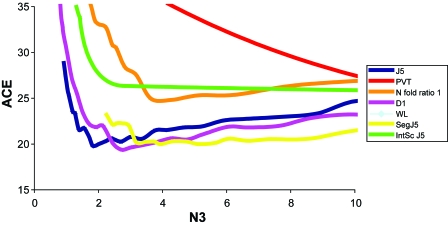
Test ACE for tests using Beer dataset As can be seen, the best ACE (19.4%) was found using the D1 test at around 3–4 genes. The second and third performers were also from the J5 family, which was consistent with Efficiency Analysis results.

**Figure 9:**
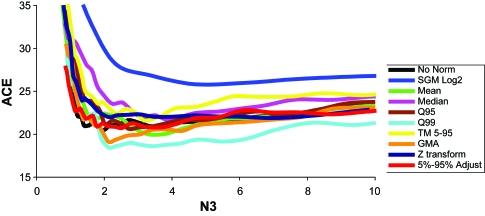
RRV estimated test ACE under various normalization methods for the Beer dataset The D1 test was performed with all transformation/normalization methods. Also consistent with Efficiency Analysis is that the Quantile 99 method again produces the most efficient results (18.45%) in combination with D1.

The training and test error results using RRV (10 resampling iterations) for the Beer dataset also indicated that D1 might be most useful. The D1 (J5 family) test appeared to produce the lowest test Achieved Classification Error (ACE) requiring only 4 genes, at a threshold of 31.0 (see Appendix D). The test ACE was ~19% using the D1 alone (Fig. 8, Appendix B10) and improved to 18.4% with the Quantile 99 normalization method (Fig. 9, Appendix B11). Importantly, both the test and the normalization methods were predicted to be most efficient! The threshold value with maximum overlap at N3≪N was used without RRV for classification of individual samples for each test. The results were as follows: A between-mean array correlation after normalization of 0.984, the between array coefficient of variation (COV) of 0.034 before normalization, and 0.062 after normalization, and a confounding index (CI; compares the average within-group correlation to the average between-group correlation; The CI should be as close to 1.0 as possible, values higher than 1.0 may indicate incidental confounding in the experimental design^21^) of 1.007 both before and after normalization. The combined score values from the four genes correctly classified 84.8% of the samples correct on average, with a sensitivity of 0.47 and a specificity of 0.942. For the PACE Analysis, the D1 test with Quantile 99 normalization and a threshold of 31 was used. 100 iterations were performed over a threshold of 0–15 with a step of 0.5. The findings were not significant based on PACE Analysis (Appendix B14).

##### Bhattacharjee external validation dataset

The data from only the four genes (Appendix B15) of interest from the Beer dataset were retained from the Bhattacharjee dataset, and the classification performed without normalization. The ACE, sensitivity, and specificity can be found in Appendix C, Table B. The best externally valid results were as follows: 79.7% of samples were classified correctly, with a sensitivity of 0.352, and a specificity of 0.942. Again, PACE Analysis was performed with the D1 test, Quantile 50 normalization, over a range of 0–10 with a step of 0.5. PACE Analysis of the test set again appeared not to be significant (Appendix B14). Xi et al.^35^ examined the same datasets for classification by lymph node status using PAM.^36^ The Beer dataset was used for training, utilizing 318 genes, and Bhattacharjee was used as an external validation set, as we have done here. They reported test lymph node positive accuracy at 94.1%, but a lymph node negative accuracy of only 21.2%, and an overall test classification accuracy of 39.1% for the Bhattacharjee dataset.

#### Classification of the Bhattacharjee dataset

The same methods and techniques were performed for cross-validation of the datasets.

##### Initial training using Bhattacharjee dataset

**Figure 10:**
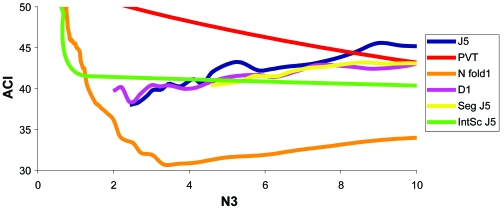
Test ACE for tests using Bhattacharjee As can be seen, the best ACE (30.7%) was found not using the D1 test, but the N fold ratio test. The J5 and D1 performed second and third best for this data set at 3–4 genes. This finding is not consistent with Efficient Analysis.

**Figure 11:**
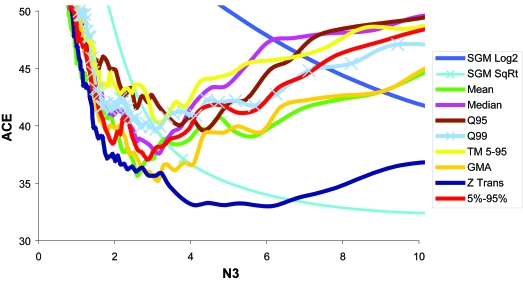
RRV estimated ACE scores for various normalizations The N fold ratio test was performed with all transformation/normalization methods. The SGM-SqRt produced the most efficient results in combination with N fold ratio (however not at the same N3 as before).

Random Resampling Validation (RRV) was performed on the Bhattacharjee et al. dataset with the same parameters as before. The results (test ACE) without and with normalization are shown in Figures 10 and 11, respectively. QC statistics were: between-mean array correlation after normalization of 0.996, the between array coefficient of variation (COV) of 0.028 before normalization, and 0.0030 after normalization, and a confounding index (CI) of 0.997 before and 0.998 after normalization. The lowest Achieved Classification Error (ACE) was 32.0% (Fig. 10, Appendix B12), was achieved using the N fold Ratio test, with six retained genes, at a threshold of 0.37 (see Appendix C). This threshold was then used without RRV to classify individual samples. The combined score values from the six genes correctly classified 73.9% of the samples correctly, with a sensitivity of 0.235 and a specificity of 0.903. For the PACE Analysis, the N fold ratio test with SGM-Square Root normalization (Fig. 11, Appendix B13) and a threshold of 0.34 was used. 100 iterations were performed over a threshold of 0–10 with a step of 0.5. The PACE Analysis appeared to show findings that are not significant (Appendix B16).

##### Beer external validation dataset

The data from only the six returned genes (Appendix B17) from the Bhattacharjee dataset were removed from the Beer dataset, and the classification performed with all normalizations. The externally valid results were as follows: 70.9% of samples were classified correctly, with a sensitivity of 0.411, and a specificity of 0.782. Again, PACE Analysis was performed with the N fold ratio test and SGM-Square Root normalization (as well as SGM-Log 2, not shown), over a range of 0–8 with a step of 0.5. PACE Analysis of the test set again seemed to show not significant findings (Appendix B16). Additionally, the D1 test was performed with and without normalization and produced very similar results to the N fold ratio classification. Xi et al.^35^ reported the following findings: a test lymph node classification error of 70% for pathology-positive, and 38% for pathology-negative patients, and an overall cross-validated accuracy of 54%. Note that here the Bhattacharjee dataset was used for training, utilizing 318 genes, and Beer was used as an external validation set.

## Discussion

Microarray technologies generate large volumes of potentially useful information and there have been many papers that have described tools and approaches for data analysis. Unfortunately the relative performance of the methods implemented, in the majority of available tools have not been compared extensively. We report on Efficiency Analysis, which we envision could possibly be an early step in a standardizing procedure for micro-array data analysis. The Efficiency Analysis described in this paper is based on the assumption that the most consistent test will provide the greatest amount of overlap at a fixed number of genes. We leave open the question of where the comparisons of the curves might be most meaningful, because this may depend entirely on the biology of a given study or investigation. An important advantage of this approach is that the identity of the genes are not examined in the comparison of different methods, thereby removing any possibility of imposing a biological bias in favoring one result over others.

Choe et al. (2005) used a control dataset to evaluate different methods. Their study was more focused on combinations of normalization/transformation/tests for differential expression, but really only compared three tests for differential expression (t-test, Cyber T, and SAM).^37^ We have shown that if the appropriate test is chosen first, transformations/normalizations may alter the accuracy of the results but not substantially. The only real difference is that our method chooses and assesses the test first, where Choe’s method follows a more classical progression of background correction to normalization to choosing the test last. By assessing the test at the beginning of the process, biases or other complications arising due to background subtraction, transformation/normalization, and/or other variability may be avoided. Thus no “standard” method of data analysis currently exists and no approach has been described for determining the most efficient method for analysis of a given dataset.

The success of any research lies on reproducibility of results, as does Efficiency Analysis. Dividing the dataset into additional variable groups a number of times had no significant effect on the results obtained (data not shown), suggesting that the Efficiency Analysis using the D1 test was robust. There is an issue of intensity-related bias in some tests for a certain number of genes, and this factor may impact Efficiency Analysis. The Segmented J5 and Intensity Scaled J5 were created to further study, and may alleviate that problem (Appendix B3). By looking at the dataset over pre-assigned quantiles, the bias is addressed. The effects of this bias are evidently minimized by the Intensity Scaled J5 test (Appendix B3), and do not significantly affect the results. The variation in performance among the different tests that we see has profound implications for studies that seek to determine whether a unique gene set might exist for a given clinical diagnosis, such as in breast cancer, and for metaanalysis.^38^ Any metaanalysis of microarray data that simply uses the t-test, for example, without attempting to determine first which of the scores of tests for finding differentially expressed genes might provide the highest true positive rate will be prone to find vast differences among studies.

In this study, only single normalization methods were considered (no combinations). There very well may be a combination of transformation/normalization methods that would produce better results than any of those tested in this study. Also, Naïve Bayes was the only classifier tested in our study. It may be that other classifiers will produce even better accuracy (e.g. PAM^36^, SVM^39^, CART^40^, etc.)

The classification results were considered taking a combination of performance characteristics and parameters into account (number of samples classified correctly, sensitivity, specificity, and number of genes). Classifying one test as the most consistent could vary depending on the parameter that is considered the most important. For our purposes, a minimal gene number, a low percent of classification error, high sensitivity and specificity were all considered in deciding which methods produced the ‘most consistent’ classification. We recognize that there are many biological factors (e.g. age, smoking history, weight, sex, diet, and environmental exposures), each of which could have influenced the performance of the test significantly. We only stratified the cases into neoplastic and non-neoplastic groups, and did not take into consideration the potential effect of influences such as those listed above, or the presence of other complex diseases. Also, there very well could be misclassification of the samples involved, of which we are unaware.

There is disappointment that accompanies the classification results not being significant according to PACE Analysis. Compared to other analyses using PAM^36^ (i.e. Xi et al. 2005), who achieved impressive classification errors (training accuracy of 88.4% when compared to pathology), we find our RRV results reassuring in that they were predicted by Efficiency Analysis. It may simply not be possible to differentiate normal lung tissue and adenocarcinoma tissue based solely on a few genes with the data and samples that were used. Xi et al. used a 318-gene set, with generally the same samples (15 omitted from the test set), to discriminate samples based on lymph node status and although the training errors were significant, the overall test validation accuracy was 39.1% (Beer-train, Bhattacharjee-test) and a cross-validated 54% (Bhattacharjee-train, Beer-test). In our study, the final classifications were the product of the test/normalization combination that produced the lowest test classification error for the existing methods. The several genes that appear to be consistently differentially expressed may be of interest in lung adenocarcinoma. Three of the genes of interest appear to be closely related which will lead to further investigation. TFF3, CALCA, and PCSK1 all are listed by Bhattacharjee et al. as genes of interest in cluster C2 (an adenocarcinoma subclass). Of the remaining genes, only HLA-B appeared as being significant in other lung carcinoma papers investigated.^9^,^13^,^41^,^42^

Sample and patient classification is an important goal. We are encouraged by Efficiency Analysis’ ability to somewhat determine the “best” performing test and normalization methods to use for classification in a way that is independent of generating the classification, with the Beer dataset. For the Bhattacharjee dataset, N fold ratio produced the best classification using RRV, but it is important to note that when D1 was independently used for classification it produced very similar results. D1 and J5 ranked second and third. When compared to the Beer dataset, the Bhattacharjee dataset appeared to be ‘less consistent’. This may be due to experimental or biological effects, but we cannot say for certain. Obviously further investigation is required. External Efficiency Analysis is available through the Overlap4 tool, and internal Efficiency Analysis is automated in caGEDA under “Computational Validation” options. We invite others to participate in using caGEDA and Efficiency Analysis using their own datasets.

During the preparation of this manuscript, the Rat toxicogenomic MAQC study was released in Nature Biotechnology.^28^ Guo et al. assessed the concordance in inter-site and cross-platform comparisons and the impact of gene selection methods on the reproducibility of profiling data in terms of differentially expressed genes.^28^ In order to validate their findings, the authors used percent overlap of gene lists, between different laboratories and/or platforms, as their evaluation method. They concluded the same as we did (prior to reading their report) that, gene lists generated by fold-change were more reproducible than those obtained by t-test or SAM.^28^ Additionally, conclusions reported by Guo et al. further support our claims as well: 1) Criteria used to define differentially expressed genes can have a dramatic impact on the overlap of the resulting genelists, 2) Findings are reproducible across laboratories and platforms when the preferred gene selection criteria are used, 3) Normalization methods do not alter the gene lists unless a p-value criterion is involved in gene selection, 4) Fold-change performed the best, followed by SAM and lastly the t-test (with mention of the problems with gene selection methods based solely on t-test p-values), 5) The importance of appropriate data analysis procedure as a whole (and may we add the order).^28^

Efficiency analysis was performed on the Rat toxicogenomic study data, the results of which can be seen in Appendix E. In support of our previous conclusions, the J5 performed best overall, better than fold-change, and the t-test performed poorly in almost all situations. We believe that including an analysis of the MAQC Rat toxicogenomic data should clarify some doubt as to whether Efficiency Analysis’ has ability to predict the ‘best test’. Although the results were minimally platform-dependent, we believe that our previous assertions are further supported by these results. We extend their findings and add that under a similar criterion, the J5 was more reproducible than fold change.

Our interpretation of the results must be stated with important caveats. Absent independent validation, for example, by RT-PCR, it may be premature to conclude the relative order of the reproducibility and consistency of the methods explored.

In conclusion, Efficiency Analysis appears to distinguish among tests that are incapable of providing consistent results and seems to also be able to generally predict which feature selection methods yield the lowest sample classification error. Although not always correct, the method does produce, if not the ‘best’ test, a general understanding of which tests yield similar internally and externally consistent results. We were surprised by the consistently poor performance of the t-test and variants of that test. We urge caution in the use of estimates of variance derived from small sample sets in high dimensions, and finding differentially expressed genes in solid tumors using tests derived from second central moment estimates.^30^ Note the J5 and related tests avoid the use of estimated variance altogether. The D1 test gave the highest percent overlap in both datasets, and although the results were validated by independent datasets, the features selected did not lead to a significant Naïve Bayes classifier, but then neither did most other gene lists from other tests. This may be due to intrinsic molecular heterogeneity within lung cancer; the Naïve Bayes model, like other fixed-marker intensity input additive linear models, uses all genes for all patients. Research is needed on classifiers that are robust to the intrinsic molecular heterogeneity of cancers.

## Figures and Tables

**Appendix B1:**
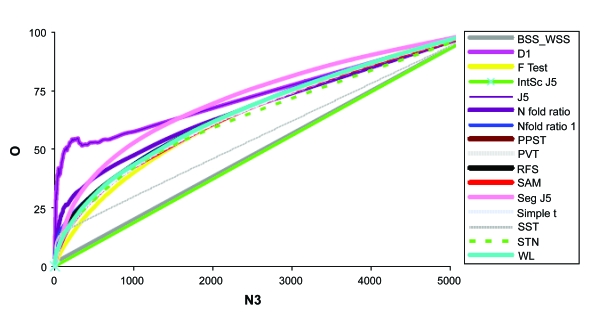
Efficiency plot of statistical tests performed without normalization The entire gene range is shown. The J5 and D1 far outperform others based on amount of overlap. The above is a comparison of O (% overlap) vs. N3 (number of genes). The results are from overlap of the Beer and Bhattacharjee significant gene lists. Abbreviations are as follows: BSS-WSS: Between Sum of Squares, Within Sum of Squares; IntSc J5: Intensity Scaled J5; N fold: [(M1−M2)/M2]; N fold Ratio 1: Ratio of Mean; PPST: Permutation Percentile Separability; PVT: Pooled Variance t Test; RFS: Random Feature Selection; SAM: Significance Analysis of Microarray; SegJ5: Segmented J5; SST: Simple Separability Test; SN: Signal to Noise Ratio; WL: Wilks Lambda Test.

**Appendix B2:**
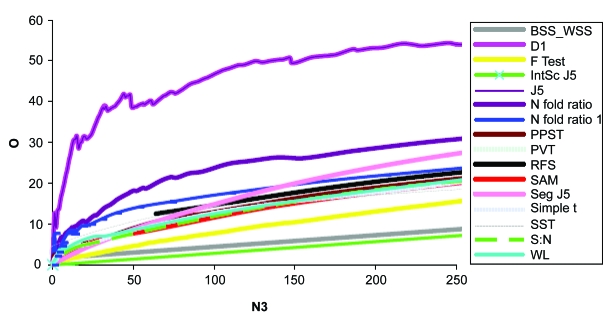
Efficiency plot of statistical tests performed without normalization A gene range of 0–250 is shown. The J5 and D1 far outperform others based on amount of overlap. The above is a comparison of O (% overlap) vs. N3 (number of genes). The results are from overlap of the Beer and Bhattacharjee significant gene lists. Abbreviations are as follows: BSS-WSS: Between Sum of Squares, Within Sum of Squares; IntSc J5: Intensity Scaled J5; N fold: [(M1−M2)/M2]; N fold Ratio 1: Ratio of Mean; PPST: Permutation Percentile Separability; PVT: Pooled Variance t Test; RFS: Random Feature Selection; SAM: Significance Analysis of Microarray; SegJ5: Segmented J5; SST: Simple Separability Test; SN: Signal to Noise Ratio; WL: Wilks Lambda Test.

**Appendix B3:**
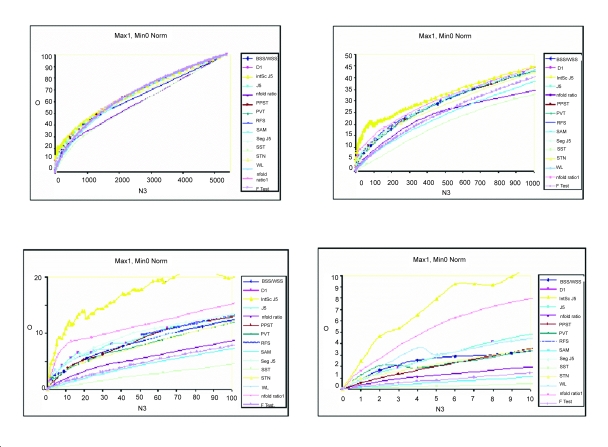
‘Pre-normalized’ plots from within gene corrected data The Intensity Scaled J5 is superior at lower number of genes. The reduction in efficiency in many tests provides evidence that the apparent bias does exist for those methods, but their relative ranking does not alter significantly the interpretation of the results.

**Appendix B4:**
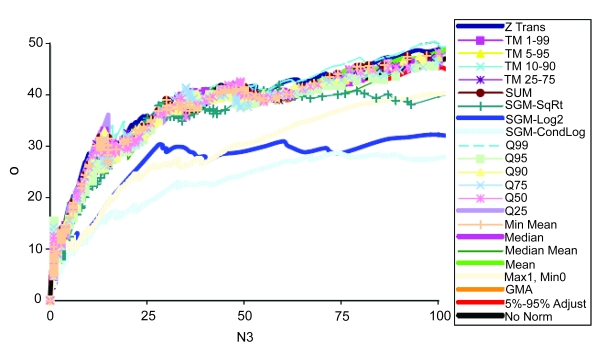
Efficiency plot of transformation/normalization methods The 0–100 gene range, comparing the Beer and Bhattacharjee datasets, is shown. Based on this figure alone it doesn’t appear that any normalization method improves the D1 efficiency. However, a closer look where N3≪m (Figure 2) reveals that a few methods actually do improve efficiency.

**Appendix B5:**
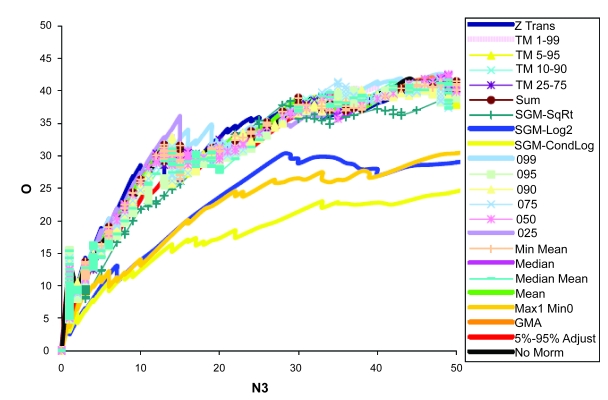
Efficiency plot of normalization and transformation methods The D1 test was used with all methods. Again, the above results are from a comparison of O (% overlap) vs. N3 (number of genes). The results are from overlap of the Beer and Bhattacharjee significant gene lists. Q: Quantile Normalization utilizing quantiles; TM: Trimmed Mean utilizing quantiles; GMA: Global Mean Adjustment; 5%–95%: Global Quantile Normalization utilizing the 5th–95th quantiles; SGM-CondLog: Subtract Global Minimum, Conditional Log Transformation. The Subtract Global Minimum procedure was performed to eliminate negative values and allow for log and square root.

**Appendix B6:**
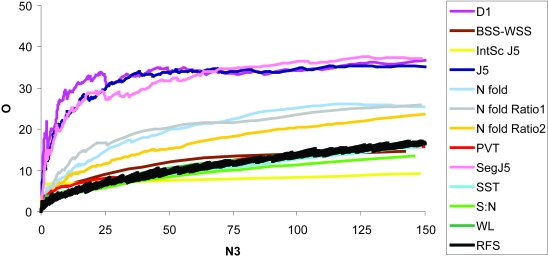
Efficiency plot of statistical tests performed without normalization for the Beer dataset Only the 0–150 gene range is shown. Certain tests appear to be more consistent depending on the number of genes desired, but a few outperform all others over the given range. The above results are from a comparison of O (% overlap) vs. N3 (number of genes). The results are from overlap of the Beer significant gene lists. O is percent overlap; N3 is the number of genes.

**Appendix B7:**
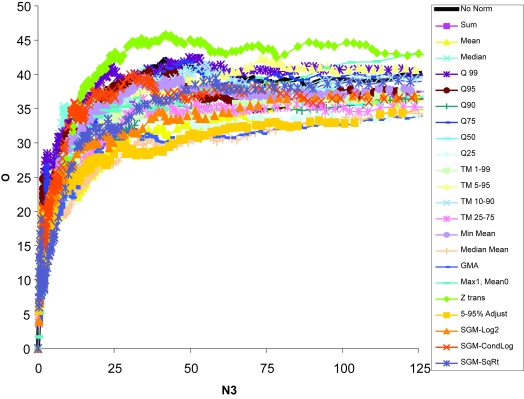
Efficiency plot of normalization and transformation methods for the Beer dataset The above results are from a comparison of O (% overlap) vs. N3 (number of genes). The results are from overlap of the Beer significant gene lists. The D1 test was used with all available methods. It is apparent that the Z transformation appeared to produce the greatest overlap over most of the given range (0–125 genes), but others appeared optimal at the extreme low end.

**Appendix B8:**
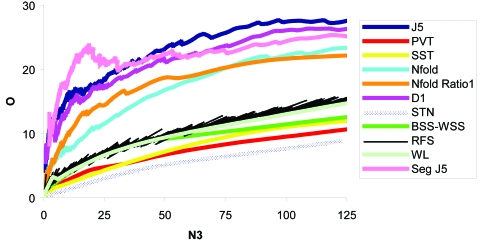
Efficiency plot of statistical tests performed without normalization for the Bhattacharjee dataset The above results are from a comparison of O (% overlap) vs. N3 (number of genes). The results are from overlap of the Bhattacharjee significant gene lists. Only the 0–125-gene range is shown. The J5 family of tests appeared to be most efficient.

**Appendix B9:**
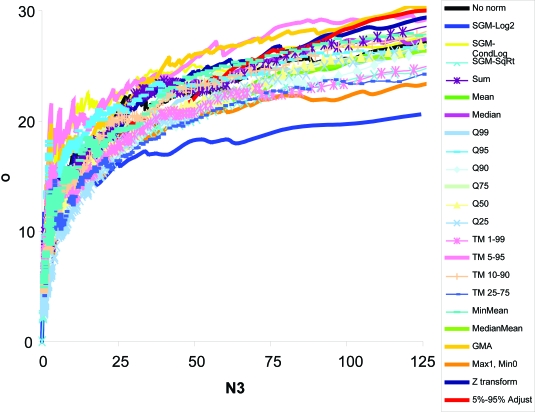
Efficiency plot of normalization and transformation methods for the Bhattacharjee dataset The above results are from a comparison of O (% overlap) vs. N3 (number of genes). The results are from overlap of the Bhattacharjee significant gene lists. The D1 test was used with all normalization methods. The Subtract Global Minimum procedure was performed to eliminate negative values and allow for log and square root transformations. At 100 genes, TM 5%–95% was the best normalization method.

**Appendix B10:**
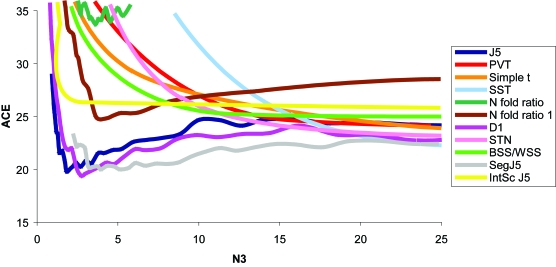
Test ACE for tests using Beer dataset As can be seen, the best ACE (19.4%) was found using the D1 test at around 3–4 genes. The second and third performers were also from the J5 family, which was consistent with Efficiency Analysis results.

**Appendix B11:**
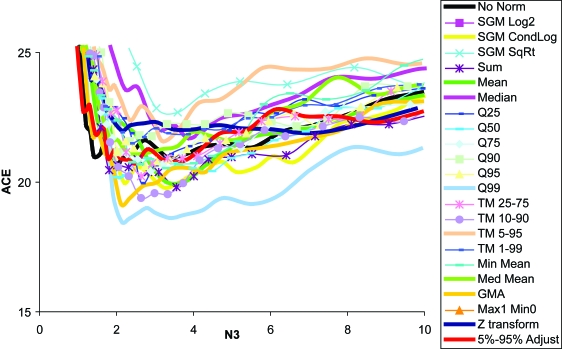
RRV estimated ACE under various normalization methods The D1 test was performed with all transformation/normalization methods. Also consistent with Efficiency Analysis is that the Quantile 99 method again produces the most efficient results (18.45%) in combination with D1.

**Appendix B12:**
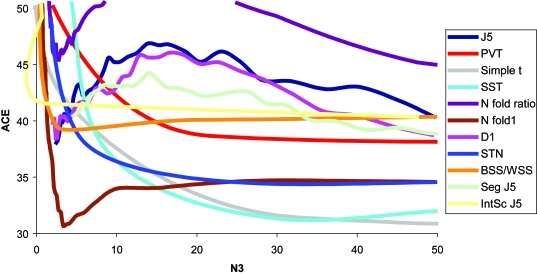
Test ACE for tests using Bhattacharjee As can be seen, the best ACE (30.7%) was found not using the D1 test, but the N fold ratio test. The J5 and D1 performed second and third best for this data set at 3–4 genes. This finding is not consistent with Efficient Analysis.

**Appendix B13:**
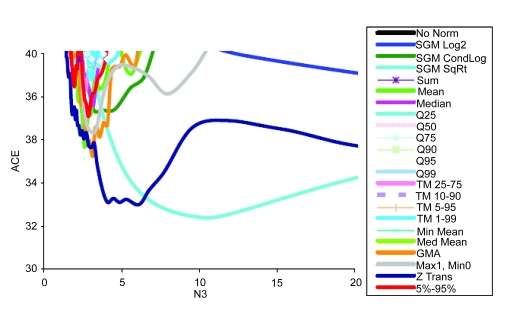
RRV estimated ACE scores for various normalizations from the Bhattacharjee dataset The N fold ratio test was performed with all transformation/normalization methods.

**Appendix B14:**
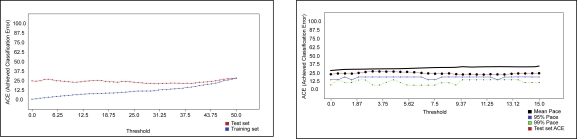
Training set Random Resampling Validation and PACE plots The lowest classification error, for the Beer dataset, was 18.4% at a threshold of 31.0, requiring only 4 genes on average. PACE Analysis showed that the findings did not appear to be significant. This is made apparent by the red line (black beaded line) never intersecting the blue 95% line.

**Appendix B15:**
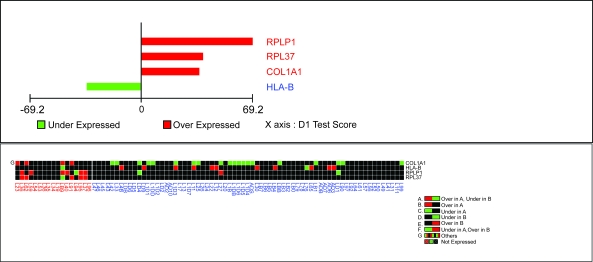
Score histogram and Expression Grid plot of significant genes found using the D1 score at a threshold of 31.0 Results are from the Beer dataset. The score histogram shows the gene regulation based on the test score, the expression pattern grid is a heat map of expression values over each sample, separated by class.

**Appendix B16:**
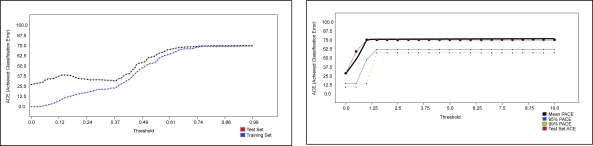
Training set Random Resampling Validation and PACE plots The best achieved classification error, for the Bhattacharjee dataset, was 32.0% at a threshold of 0.37, requiring five genes. The PACE Analysis shows that the findings did not seem significant. This is made apparent by the red line (thick black line) never falling below the blue 95% line.

**Appendix B17:**
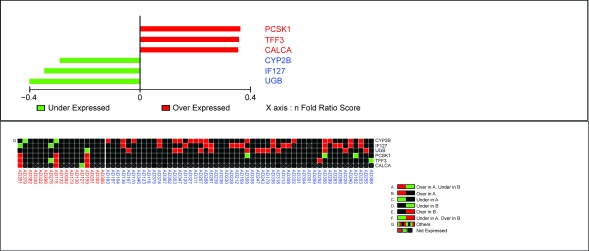
Score histogram and Expression Grid plot of significant genes found using the N fold Ratio score at a threshold of 0.37 Results are from the Bhattacharjee dataset.

**Appendix E1:**
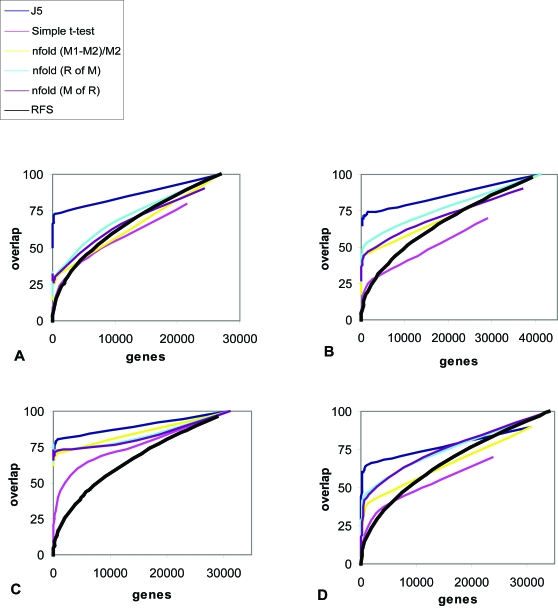
Efficiency plot of kidney data from the Rat toxicogenomic study **A**) ABI C-AA, **B**) Agilent C-AA, **C**) Affymetrix C-AA, **D**) GE C-AA. The J5 appears to perform the best over all platforms used. Dark blue: J5; pink: simple t-test; yellow: nfold1; turquoise: nfold2; purple: nfold3; black: random feature selection. AA = Aristolochic acid.

**Appendix E2:**
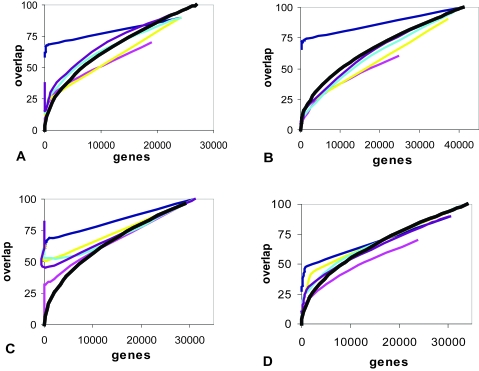
Efficiency plot of liver data from the Rat toxicogenomic study. **A**) ABI C-AA, **B**) Agilent C-AA, **C**) Affymetrix C-AA, **D**) GE C-AA. The J5 appears to perform quite well compared to the other methods. Dark blue: J5; pink: simple t-test; yellow: nfold1; turquoise: nfold2; purple: nfold3; black: random feature selection. AA = Aristolochic acid.

**Appendix E3:**
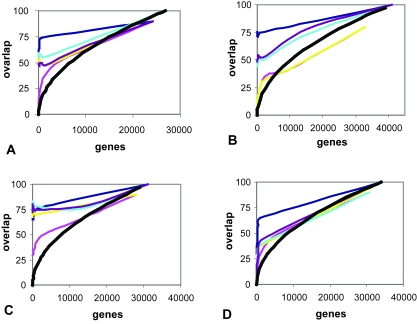
Efficiency plot of liver data from the Rat toxicogenomic study **A**) ABI C-C, **B**) Agilent C-C, **C**) Affymetrix C-C, **D**) GE C-C. The J5 appears to again perform quite well compared to the other methods. Dark blue: J5; pink: simple t-test; yellow: nfold1; turquoise: nfold2; purple: nfold3; black: random feature selection. C-C = Comfrey roots.

**Appendix E4:**
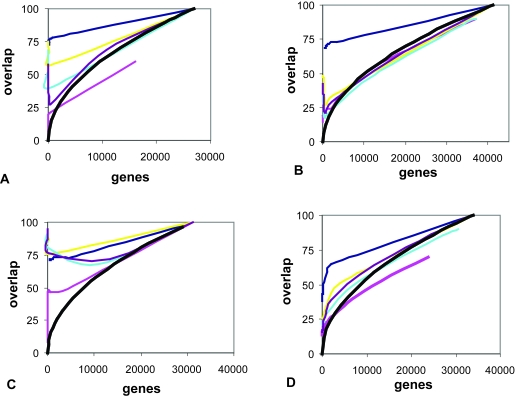
Efficiency plot of liver data from the Rat toxicogenomic study **A**) ABI C-R, **B**) Agilent C-R, **C**) Affymetrix C-R, **D**) GE C-R. The J5 appears to perform the best in 3 out of 4 platforms (only being outperformed by nfold1 on the Affymetrix platform). Dark blue: J5; pink: simple t-test; yellow: nfold1; turquoise: nfold2; purple: nfold3; black: random feature selection. C-R = Comfrey Roots/Riddelliine.

## Appendix A

The following descriptions taken from the caGEDA website:

### Threshold-based tests

J5: compares the difference of means in any gene to the average difference in means over the whole array.

D1: performs the J5 once, removes all significant genes, and performs the J5 again to capture any genes that were missed initially.

Signal-to-Noise: similar to t test with a high positive false discovery rate.

Segmented J5: developed out of concern over intensity-related bias in the tests. The segmented J5 was designed to determine whether the J5 test was more efficient than the other tests when the denominator was calculated only using genes with similar intensity. In the segmented J5, genes are ranked by median intensity and a number of quantiles are determined. The mean of the absolute value of the difference of means is used for each gene within a quantile as the J5 denominator.

Intensity Scaled J5: similar to segmented J5 but scaled using the entire range of intensities.

### Normalization methods

#### Within-array

Sum: all intensity values on the array are multiplied by the sum of all intensity values on the array.

Mean: all intensity values on the array are multiplied by the mean of all intensity values on the array.

Median: all intensity values on the array are multiplied by the median of all intensity values on the array.

Quantile/Percentile: all intensity values on the array are multiplied by the mean of all intensity values on the array beyond the specified quantile.

Trimmed mean: all intensity values on the array are multiplied by the mean of all intensity values on the array between two specified quantiles.

#### Among-array

Median mean: the mean intensity of each array is calculated, the median mean is identified, and an array-specific multiplicative factor is defined as the ratio of that array’s mean to the median mean

Minimum mean: same as median mean but instead of normalizing to the median mean, the scaling factor is the minimum mean of all of the arrays.

Global mean adjustment: the global mean intensities of all arrays are calculated, then the difference between each individual array mean and the global mean is calculated, the array-specific difference value is then added to or subtracted from each individual expression intensity value on an array and the result is that all arrays now have the same overall mean.

Max1, Min0: re-scales each expression intensity profile so the array-specific maxima and minima are equal in all arrays.

Z-transformation: similar to log transformation in that it can change the shape of the distribution, but can reduce performance of some tests for differentially expressed genes.

## Appendix C

**Table A:** Genelist from efficiency analysis of both datasets % found = percentage of time that the gene was found on all gene lists from all data splits.

	Beer-	GenBank		#	Bhattacharjee-Gene	GenBank			Bhattacharjee-Gene	GenBank	
#	Gene	ID	% found			ID	% found	#		ID	% found
1	ALDOA	NM_000034	100	1	ALDH1	NM_000689	100	44	CEBPB	NM_005194	66.666
2	COL1A1	NM_000088	100	2	ASCL1	NM_004316	100	45	CLIC1	NM_001288	66.666
3	COL5A2	NM_000393	100	3	B2M	NM_004048	100	46	MMP11	NM_005940	66.666
4	GAPD	NM_002046	100	4	BGN	NM_001711	100	47	MSN	NM_002444	66.666
5	JUND	NM_005354	100	5	CLU	NM_001831	100	48	APOC1	NM_001645	66.666
6	KIAA0220		100	6	COL1A2	NM_000089	100	49	BENE	NM_005434	66.666
7	LGALS1	NM_002305	100	7	CYP2B		100	50	COL3A1	NM_000090	66.666
8	MGP	NM_000900	100	8	FOXG1A	NM_005249	100	51	PFN1	NM_005022	66.666
9	PTGDS	NM_000954	100	9	FTL	NM_000146	100	52	PGAM1	NM_002629	66.666
10	RPL37	NM_000997	100	10	FXYD5	NM_144779	100	53	PTGDS	NM_000954	66.666
11	RPLP0	NM_053275	100	11	IFI27	NM_005532	100	54	COL6A1	NM_001848	66.666
12	RPLP1	NM_001003	100	12	LDHB	NM_002300	100	55	RPL18		66.666
13	RPS23	NM_001025	100	13	MDK	NM_001012334	100	56	RPL27A	NM_000990	66.666
14	S100A10	NM_002966	100	14	MIF	NM_002415	100	57	RPL29	NM_000992	66.666
15	S100A11	NM_005620	100	15	NBL1	NM_182744	100	58	RPL3		66.666
16	S100A9	NM_002965	100	16	NK4	NM_001012631	100	59	APOE	NM_000041	66.666
17	S100P	NM_005980	100	17	PCSK1	NM_000439	100	60	CRIP1	NM_001311	66.666
18	SLPI	NM_003064	100	18	RNASE1	NM_002933	100	61	RPS15A	NM_001019	66.666
19	SPARC		100	19	RPLP1	NM_001003	100	62	CYBA	NM_000101	66.666
20	TMSB4X	NM_021109	100	20	RPS10	NM_001014	100	63	RPS20	NM_001023	66.666
21	VIM	NM_003380	100	21	RPS2	NM_002952	100	64	RPS21	NM_001024	66.666
22	CST3	NM_000099	66.666	22	RPS24	NM_033022	100	65	ACTG1	NM_001614	66.666
23	OAZ1	NM_004152	66.666	23	S100A10	NM_002966	100	66	DIA4	NM_000903	66.666
24	CSTB	NM_000100	66.666	24	SEPW1	NM_003009	100	67	S100A8	NM_002964	66.666
25	RPL18	NM_000979	66.666	25	SHC1	NM_003029	100	68	SELENBP1	NM_003944	66.666
26	RPL3	NM_000967	66.666	26	SLPI	NM_003064	100	69	DUSP4	NM_057158	66.666
27	EEF2	NM_001961	66.666	27	SPARC	NM_003118	100	70	SFTPB	NM_000542	66.666
28	FN1	NM_212476	66.666	28	SPP1	NM_001040058	100	71	SFTPD	NM_003019	66.666
29	ACTB	NM_001101	66.666	29	TAGLN2	NM_003564	100	72	EIF4G2	NM_001418	66.666
30	RPS11	NM_001015	66.666	30	TFF3	NM_003226	100	73	EPHX1	NM_000120	66.666
31	HE1	NM_006432	66.666	31	TMSB10	NM_021103	100	74	FLJ20493	NM_019051	66.666
32	RPS28	NM_001031	66.666	32	YWHAH	NM_003405	100	75	SPINK1	NM_003122	66.666
33	RPS8	NM_001012	66.666	33	COL6A2	NM_001849	88.888	76	FOSB	NM_006732	66.666
34	HLA-B	NM_005514	66.666	34	BF	NM_001710	77.777	77	BIG2	NM_006420	66.666
35	HLA-DRA	NM_019111	66.666	35	H2AFO	NM_003516	66.666	78	FTH1	NM_002032	66.666
36	HLA-DRB1	NM_02124	66.666	36	HE4	NM_006103	66.666	79	TGFBI	NM_000358	66.666
37	IGKC		66.666	37	HLA-A	NM_002116	66.666	80	C1QB	NM_000491	66.666
38	SFTPB	NM_198843	66.666	38	HSPD1	NM_199440	66.666	81	UGB	NM_003357	66.666
39	SFTPD	NM_000542	66.666	39	CCND1	NM_053056	66.666	82	VIM	NM_003380	66.666
40	COL1A2	NM_000089	66.666	40	IGFBP3	NM_001013398	66.666	83	CALCA	NM_001741	66.666
41	COL3A1	NM_000090	66.666	41	IGL@		66.666	84	GPX4	NM_002085	55.555
42	TAGLN2	NM_003564	66.666	42	CEACAM6	NM_002483	66.666	85	LTA4H	NM_000895	55.555
				43	LGALS1	NM_002305	66.666	86	TEGT	NM_003217	55.555

**Table B:** Bhattacharjee dataset used for external validation of the beer dataset classifier.

Norm method	% Correct	Sensitivity	Specificity	Between-mean array correlation(after normalization)	Between array coefficient of variation (after normalization)	Confounding index (after normalization)
Quantile 50	0.797	0.352	0.942	0.993	0.207	0.994
SGM-Log2	0.782	0.176	0.98	0.999	0.069	0.998
Max1, Min0	0.768	0.058	1	0.998	0.126	0.994
SGM-Square Root Trans	0.768	0.058	1	0.999	−1.103	0.999
5%–95% Adjust	0.753	0	1	0.998	0.173	0.994
SGM-Cond Log Trans	0.753	0.058	0.98	0.999	0.38	1
Global Mean Adjust	0.753	0	1	0.999	0	0.994
Mean	0.753	0.117	0.961	0.997	0	0.994
Minimum Mean	0.753	0.117	0.961	0.997	0	0.994
Quantile 25	0.753	0.117	0.961	0.997	0.398	0.994
Quantile 75	0.753	0.117	0.961	0.998	0.154	0.994
Quantile 90	0.753	0.117	0.961	0.998	0.154	0.994
Quantile 95	0.753	0.117	0.961	0.998	0.154	0.994
Quantile 99	0.753	0.117	0.961	0.998	0.154	0.994
Sum	0.753	0.117	0.961	0.997	0	0.994
Median	0.739	0.117	0.942	0.994	0.178	0.994
Median Mean	0.739	0.058	0.961	0.999	0.503	0.994
No Norm	0.739	0	0.98	0.999	0.246	0.994
Trimmed Mean 10–90	0.739	0.235	0.903	0.994	0.218	0.994
Trimmed Mean 1–99	0.739	0.235	0.903	0.994	0.218	0.994
Trimmed Mean 25–75	0.739	0.117	0.942	0.994	0.178	0.994
Trimmed Mean 5–95	0.739	0.235	0.903	0.994	0.218	0.994
Z Trans	0.724	0	0.961	0.998	−15.474	0.994

**Table C:** Beer dataset used for external validation of the Bhattacharjee dataset classifier.

Norm Method	% Correct	Sensitivity	Specificity	Between-mean array correlation (after normalization)	Between array coefficient of variation (after normalization)	Confounding index (after normalization)
SGM-Log2	0.709	0.411	0.782	0.931	0.105	0.985
Quantile 75	0.511	0.647	0.478	0.996	0.807	0.989
Mean	0.488	0.705	0.434	0.993	0	0.989
Minimum Mean	0.488	0.705	0.434	0.993	0	0.989
Sum	0.488	0.705	0.434	0.993	0	0.989
Z Trans	0.476	0.647	0.434	0.992	−30.447	0.989
5%–95% Adjust	0.465	0.882	0.362	0.991	0.252	0.989
Global Mean Adjust	0.465	0.705	0.405	0.902	0	0.989
Max1, Min0	0.465	0.882	0.362	0.991	0.258	0.989
Quantile 90	0.465	0.647	0.42	0.991	0.265	0.989
Quantile 95	0.465	0.647	0.42	0.991	0.265	0.989
Quantile 99	0.465	0.647	0.42	0.991	0.265	0.989
SGM-Square Root Trans	0.441	0.941	0.318	0.961	0.372	0.988
No Norm	0.43	1	0.289	0.902	0.862	0.989
Trimmed Mean 10–90	0.418	0.764	0.333	0.987	0.767	0.989
Trimmed Mean 1–99	0.418	0.764	0.333	0.987	0.767	0.989
Trimmed Mean 5–95	0.418	0.764	0.333	0.987	0.767	0.989
Median Mean	0.406	1	0.26	0.557	2.067	0.989
Quantile 25	0.406	1	0.26	−0.721	−10.2	1.018
SGM-Cond Log Trans	0.36	1	0.202	0.966	0.886	1.026
Quantile 50	0.36	0.941	0.217	0.622	2.417	0.989
Trimmed Mean 25–75	0.36	0.941	0.217	0.768	1.915	0.989
Median	0.348	0.941	0.202	0.694	2.2	0.989

## Appendix D

### Genes of interest

The genes and descriptions are described below. The information was taken from The NCBI Entrez nucleotide database (http://www.ncbi.nlm.nih.gov/entrez) using the corresponding GenBank ID’s.

**RPLP1** (NM_001003, Ribosomal protein, large, P1, transcript variant 1). (AKA: P1, RPP1, FLJ27448).

This gene encodes a ribosomal phosphoprotein that is a component of the 60S subunit and is located in the cytoplasm. It plays an important role in the elongation step of protein synthesis. The encoded protein is acidic, and its functions include RNA binding and being a structural constituent of ribosome. RPLP1 is involved in the protein biosynthesis and translational elongation processes.

**RPL37** (NM_000997, Ribosomal protein L37) (AKA: MCG99572).

This gene encodes a ribosomal protein that is a component of the 60S subunit, and is located in the cytoplasm. The protein contains a C2C2-type zinc finger-like motif.

**COL1A1** (NM_000088, Collagen, type I, alpha 1) (AKA: OI4).

This gene encodes the major component of type I collagen, found in most connective tissues, and the only component of the collagen found in cartilage. Translocations in this region may result in unregulated expression of growth factor.

**HLA-B** (NM_005514, Major histocompatibility complex, class I, B).

This class I molecule is a heterodimer (beta-2 microglobulin). Class I molecules play a central role in the immune system by presenting peptides derived from the ER lumen. Class I molecules are expressed in nearly all cells. Polymorphisms within this gene are responsible for the peptide binding specificity of each Class I molecule. HLA-B is integral to plasma membrane, and functions as a Class I receptor. HLA-B is involved in antigen processing and presentation in the immune response. HLA-B, (MHC class 1, B receptor) was found in the Beer et al. paper as one of the top 100 survival-related genes from cross-validation, found to be significantly (p = 0.000391) downregulated in Stage 3 vs. Stage 1 by 40%, but not significantly different in normal vs. tumor.

**PCSK1** (NM_000439, Proprotein convertase subtilisin/kexin type 1) (AKA: PC1, PC3, NEC1, SPC3).

Proprotein convertases process precursor proteins into their biologically active products. PCSK1 is a Type I proinsulin-processing enzyme that plays a role in regulating insulin biosynthesis. Mutations in this gene are thought to contribute to obesity, and this protein has also been associated with carcinoid tumors. PCSK1 is listed on the marker gene list by Bhattacharjee as a member of their C2 cluster subclass.

**TFF3** (NM_003226, Trefoil factor 3 (intestinal)) (AKA: ITF, TFI, HITF).

Members of the trefoil family are secretory proteins expressed in intestinal mucosa. They may protect the mucosa from insults, stabilize the mucus layer and affect healing of the epithelium. This gene is expressed in goblet cells of the intestines and colon. TFF3 is listed on the marker gene list by Bhattacharjee twice as a member of the C2 cluster subclass.

**CALCA** (NM_001741, Calcitonin/calcitonin-related polypeptide, alpha). (AKA: CT, KC, CGRP, CALC1, CGRP1, CGRP-I).

Calcitonin is a peptide hormone synthesized by the thyroid. CALCA is also listed twice on the marker gene list by Bhattacharjee as a member of the C2 cluster subclass.

**CYP2B** (NM_000767, Cytochrome P450, family 2, subfamily B, polypeptide 6) (AKA: CPB6, IIB1, P450, CYP2B6, CYPIIB6).

CYP2B, catalyzes many reactions involved in drug metabolism and synthesis of cholesterol, steroids and other lipids. This protein is localized to the ER and is known to metabolize some xenobiotics, such as anti-cancer drugs.

**IFI27** (NM_005532, Interferon, alpha-inducible protein 27, transcript variant a) (AKA: P27, ISG12).

**UGB** (NM_003357, Secretoglobin, family 1A, member 1 (uteroglobin)). (AKA: SCGB1A1, CC10, CC16, CCSP)).
